# P-303. Mupirocin Resistance is Common in Tampa, Florida *Staphylococcus aureus* Clinical Isolates

**DOI:** 10.1093/ofid/ofae631.506

**Published:** 2025-01-29

**Authors:** Ariana Virgillio, Emily Felton, Deanna Becker, Amorce Lima, Suzane Silbert, Emily Coughlin, Lindsey Shaw, Kami Kim

**Affiliations:** USF Morsani College of Medicine, Tampa, Florida; University of South Florida, Tampa, Florida; Tampa General Hospital, Tampa, Florida; Tampa General Hospital, Tampa, Florida; Tampa General Hospital, Tampa, Florida; USF Morsani College of Medicine, Tampa, Florida; University of South Florida, Tampa, Florida; University of South Florida, Tampa, Florida

## Abstract

**Background:**

Mupirocin (Mup) is a topical drug used to decolonize carriers of *Staphylococcus aureus* (SA) to prevent later SA infection. A spot survey of SA isolates at Tampa General Hospital (TGH) revealed a prevalence in Mup resistance of ∼40%, leading to a change of decolonization protocol from mupirocin to povidone iodine in January 2020, just prior to the COVID pandemic. We determined the prevalence of Mup resistance in a random sampling of clinical isolates before and after change in decolonization protocol.

**Methods:**

A total of 211 PreCOVID (before 2020) and 173 PostCOVID (after 2021) samples from various sources (blood, bone, eye, fluid, respiratory, urine, and wound) were tested with minimum inhibitory concentration for Mup resistance using literature values for susceptible, low resistance, and high resistance. Oxacillin sensitivity, to verify MRSA isolates, was tested in parallel. Results were analyzed via χ² for significance (p=0.05).

**Results:**

Comparison of Mup resistance levels between the Pre- and PostCOVID time points showed no significant difference (p=0.460). PreCOVID: 71.1% susceptible, 8.1% low resistance, 20.9% high resistance. PostCOVID: 74.0% susceptible, 9.8% low resistance, and 16.2% high resistance. MRSA status correlated with Mup resistance in both Pre- (p< 0.001) and PostCOVID samples (p=0.001).

Blood vs Non-Blood samples differed in Mup resistance in the PreCOVID period (20.7% blood vs 34.1% non-blood, p=0.037), whereas samples in the PostCOVID period showed no significant difference in Mup resistance (22.8% blood vs 28.7% non-blood, p=0.375). Notably, 46.2% PreCOVID Respiratory were resistant to Mup (p=0.016). 26.3% PostCOVID Respiratory were resistant to Mup (p=0.651).

**Conclusion:**

Change in decolonization protocol did not influence the overall prevalence of Mup resistance. Respiratory samples made up a significant proportion of the resistant samples in the PreCOVID time point, suggesting differences in prevalence of Mup resistance in different clinical sites. Further molecular epidemiological studies are needed to determine if specific sites of infection or specific patient groups are more likely to be colonized with Mup resistant SA isolates.

**Disclosures:**

**All Authors**: No reported disclosures

